# The Tomato *BLADE ON PETIOLE* and *TERMINATING FLOWER* Regulate Leaf Axil Patterning Along the Proximal-Distal Axes

**DOI:** 10.3389/fpls.2018.01126

**Published:** 2018-08-06

**Authors:** Anat Izhaki, John P. Alvarez, Yuval Cinnamon, Olga Genin, Raya Liberman-Aloni, Yoram Eyal

**Affiliations:** ^1^Institute of Plant Sciences, Agricultural Research Organization, Volcani Center, Rishon LeZion, Israel; ^2^School of Biological Sciences, Clayton Campus, Monash University, Melbourne, VIC, Australia; ^3^Department of Poultry and Aquaculture Science, Institute of Animal Science, Agricultural Research Organization, Volcani Center, Rishon LeZion, Israel

**Keywords:** boundary zone, axillary meristem, BLADE ON PETIOLE, abscission, TERMINATING FLOWER, leaf axil, development, tomato

## Abstract

Leaf axil patterning occurs concomitantly with leaf development and takes place at the boundary zone which demarcates the initiating leaf primordium from the shoot apical meristem. Subsequent growth and differentiation result in establishment of the axillary meristem and abscission zone (AZ) along the proximal-distal axis of the leaf axil, yet the molecular mechanisms that regulate these events are poorly understood. We studied the role of the tomato *BLADE ON PETIOLE* (*SlBOP*) boundary gene family on the development of the leaf axil using *BOP*-silenced plants as well as *BOP*-mutated lines. We show that silencing of the tomato *SlBOP* gene family affects patterning of the leaf axil along the proximal-distal axis, manifested by dispositioning of the AM and abnormal development of the adjacent tissue resulting in lack of a functional leaf AZ. Dissection of the role of each of the three tomato *SlBOPs* by analysis of single, double and triple null-mutants demonstrated that *SlBOP2* is the dominant gene in leaf axil patterning, but does not rule out involvement of *SlBOP1* and *SlBOP3* in correct AM positioning. We further studied the potential role of TERMINATING FLOWER (TMF), a transcription factor which was previously shown to interact with SlBOPs, in leaf axil patterning using TMF mutant tomato lines. The results suggest that similar to SlBOP2, TMF is involved in leaf axil proximal-distal patterning and AZ development.

## Introduction

The shoot apical meristem (SAM) is established during embryogenesis and serves as the origin of plant vegetative development and the indeterminate pattern of plant growth. Vegetative growth and development occurs in the SAM by a fine balance between two types of cells – meristematic cells which maintain a non-differentiated identity, and cells which depart from the meristem and differentiate to produce organ primordia (Bowman and Eshed, 2000; Barton, 2010). The spatial proximity between the meristematic and the differentiated cells is separated by a layer of cells with a distinct entity termed the boundary zone (BZ). BZs are characterized by small cells with limited cell division and restricted proliferation (Breuil-Broyer et al., 2004; Rast and Simon, 2008). Lateral branches originate from axillary meristems (AMs), which are located on the adaxial side of each leaf axil. Differentiation of the AM takes place in BZs early in development, concomitantly with leaf initiation, and requires auxin minima achieved by polar auxin transport from the primordium toward the SAM (Wang Q. et al., 2014; Wang Y. et al., 2014; Burian et al., 2016; Wang et al., 2016).

In eudicots, leaf developmental programs also combine the formation of specialized abscission tissue within the BZ that is essential for the programmed process of lateral organ detachment, which enables plants to shed no longer needed or infected leaves. Leaf abscission occurs in designated tissues termed abscission zones (AZs), located in the leaf axil between the abscised leaf and the remaining plant body. Spatial and temporal regulation along the leaf axil proximal-distal axis is likely required to ensure the proper positioning of a proximal AM and a distal AZ, allowing leaf abscission while maintaining the AM attached to the plant body. Leaf AZ development occurs at BZs chronologically after AM and leaf primordium establishment (Addicott, 1945; Brown and Addicott, 1950; Gawadi and Avery, 1950; Osborne and Sargent, 1976; Sexton and Roberts, 1982), and yet, little is known regarding the spatial and temporal coordination and regulation of BZ, AM, and AZ differentiation and development along the proximal-distal axis during leaf axil formation in eudicots.

Boundary cells express distinct transcription factors, which repress cell proliferation and determine boundary cell fate. Since boundary tissues separate between different organs, mutations in boundary genes often lead to fusion of the adjacent organs or loss of organ initiation (Sluis and Hake, 2015). One group of boundary genes are the *BLADE ON PETIOLE* (*BOP*) gene family initially characterized in Arabidopsis loss of function mutants which form ectopic blade outgrowths along the leaf petioles (Ha et al., 2003, 2004; Hepworth et al., 2005; Norberg et al., 2005). Consistent with their role in BZs and floral organ development, *bop2* null mutants exhibit fused inflorescence organs, while *bop1 bop2* double mutants exhibit enhanced inflorescence phenotypes (Hepworth et al., 2005; Norberg et al., 2005; Ha et al., 2007). *BOP1* and *BOP2* are also expressed in the boundary of the emerging leaf primordium (Jun et al., 2010) and were shown to regulate organ primordia initiation and boundary formation along the leaf proximal-distal axis by inducing three members of the *LATERAL ORGAN BOUNDARIES* (*LOB*) domain (*LBD*) gene family; *ASYMETRIC LEAVES2* (*AS2*), LBD36 and *LOB* (Ha et al., 2007; Jun et al., 2010). The latter was shown to promote boundaries by repressing brassinosteroid signaling, a hormone which promotes cell division and expansion (Bell et al., 2012; Gendron et al., 2012). BOP1 and BOP2 also act in the BZs to repress the meristem identity genes belonging to the class I KNOTTED1-like homeobox (KNOX) family; *BREVIPEDICELLUS* (*BP*), *KNAT*2, and *KNAT*6 (Norberg et al., 2005; Ha et al., 2007; Stenvik et al., 2008; Khan et al., 2014; Žádníková and Simon, 2014; Hepworth and Pautot, 2015). In barley, the BOP homolog, *Uniculme4* (*Cul4*) was shown to be expressed in leaf axil boundaries to regulate AM differentiation and tiller development (Tavakol et al., 2015). Later in development, the *BOP* genes have been shown to control petal AZ differentiation in Arabidopsis and tobacco by promoting cell division at the corresponding abscission sites (McKim et al., 2008; Wu et al., 2012). In tomato, similar to Arabidopsis and tobacco, loss of function of the *SlBOP* genes led to loss of floral abscission (Xu et al., 2016). Thus, the emerging picture connects the boundary BOP proteins to the developmental events occurring at the BZ. However, despite the spatial and temporal relationship between leaf BZ, AM, and AZ development, function of the BOP gene family throughout leaf axil polarity establishment has not been demonstrated.

The BOP family displays high similarity to the *NONEXPRESSOR OF PATHOGENESIS-RELATED GENES*1 (*NPR*1) family, encoding BTB/POZ (Broad complex, Tramtrack and Bric-a-brac/POX virus and Zinc finger) -ankyrin proteins involved in plant defense (Ha et al., 2004, 2007; Hepworth et al., 2005; Fu and Dong, 2013). The accumulating evidence suggests that, similar to NPR1, one mode of action of BOP proteins is in regulating gene expression by interacting with different DNA-binding TFs to regulate developmental processes. BOP was shown to interact in Arabidopsis with PERIANTHIA (PAN), a TGA family TF, to regulate floral patterning (Hepworth et al., 2005). In tomato the *SlBOP* gene family was found to consist of three family members, based on similarity to Arabidopsis BOP and phylogenetic analysis (Ichihashi et al., 2014; Xu et al., 2016). *SlBOP* genes were shown to enhance the transcription-promoting activity of TERMINATING FLOWER (TMF), a weak TF belonging to the ALOG (Arabidopsis LSH1 and oryza G1) protein family, by creating TMF-SlBOPs complexes, which repress meristem maturation and regulate tomato floral patterning (MacAlister et al., 2012; Xu et al., 2016).

To study leaf axil patterning and abscission zone development in dicots, we used tomato as a model plant. The current study focuses on the role of the tomato SlBOP family in leaf axil patterning along the proximal-distal axis to specify proper AM positioning and adjacent leaf AZ development. We present evidence that *SlBOP* gene function is involved in determining the AM position at the stem-petiole junction and in regulating the development of a functional leaf AZ, suggesting dual yet related roles in leaf boundary patterning and AZ differentiation. Dissection of the role of the three tomato *SlBOPs* demonstrates that *SlBOP2* is the dominant gene of the three family members in leaf axil patterning, but does not rule out involvement of *SlBOP1* and *SlBOP3* in the process. We further show that SlBOPs may regulate leaf axil proximal-distal pattern formation in concert with the transcription factor TMF.

## Results

### Tomato *SlBOP* Function to Pattern Leaf Axil by Regulating AM Positioning and Leaf AZ Development

**FIGURE 1:**
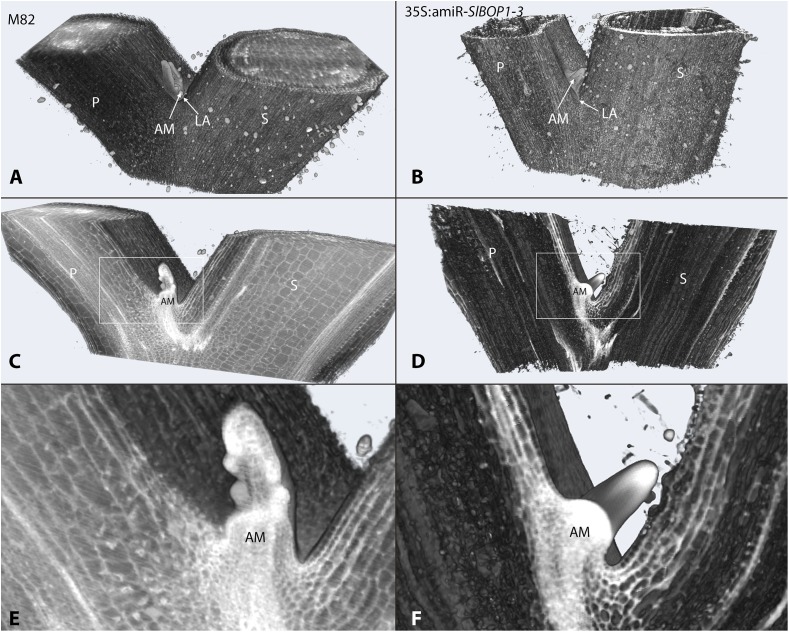
*SlBOP* downregulation leads to altered leaf axil patterning. HREM 3D reconstructed models of the first leaf axil of 4-week old wild type (M82) **(A,C,E)** and 35S:amiR-*SlBOP1-3* **(B,D,F)** plants. **A,B**, overview of the 3D HERM models of WT **(A)** and 35S:amiR-*SlBOP1-3* **(B)** plants. **C,D**, HERM models showing a cross section through WT **(C)** and 35S:amiR-*SlBOP1-3* **(D)** AM. **E,F**, close up of the leaf axil framed in **C,D**, respectively. P, Petiole; S, stem; AM, axillary meristem; LA, leaf axil.

**FIGURE 2:**
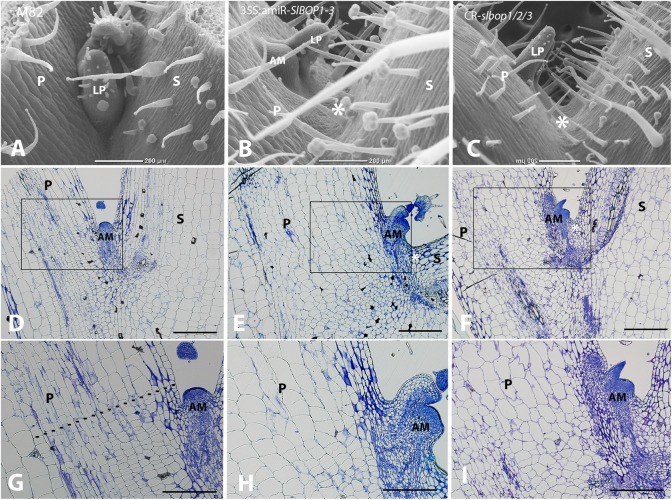
*SlBOP* downregulation and mutagenesis leads to altered leaf axil patterning. SEM analysis **(A–C)** and Histologic analysis **(D–I)** of the first leaf axil of a 4-week old wild type (M82) **(A,D,G)**, 35S:amiR-*SlBOP1-3* **(B,E,H)** and *CR-slbop* triple mutant *CR-slbop1*/2/3 **(C,F,I)**. **G–I**, close up of the leaf axil framed in **D–F**, respectively. Dotted line in **G** marks the junction between small and elongated cells. P, Petiole; S, stem; AM, axillary meristem; LP, leaf primordium; asterisks – location of AM in wild type plants. Bars **(A–F)** = 200 μm, **(G–I)** = 100 μm.

In tomato *SlBOP* genes function to regulate leaf shape and complexity (Ichihashi et al., 2014; Xu et al., 2016). Variation in *SlBOP* expression patterns were shown to account for the diversity in leaf complexity between domesticated tomato (*Solanum lycopersicum*) and its wild relatives (Ichihashi et al., 2014). Earlier function of *BOP* genes as markers of lateral organ boundaries was previously studied in Arabidopsis (Ha et al., 2004, 2007; Hepworth et al., 2005; Norberg et al., 2005; Khan et al., 2012), however, Arabidopsis lacks functional AZs and thus provides limited information regarding leaf axil patterning. In tomato *SlBOP* genes are expressed in leaf primordium BZ during early stages of leaf primordium development (Xu et al., 2016), yet, very little is known regarding their function in later stages of leaf axil patterning and AM development. We studied the role of the SlBOP family in tomato leaf axil patterning by utilizing BOP knockout and partially silenced tomato plants: (1) a gene editing based *CRISPR (CR)-slbop* triple mutant line, *CR-slbop1/2/3* (Xu et al., 2016) and (2) transgenic plants harboring an artificial micro RNA designed to downregulate all three members of the tomato *SlBOP* gene family (Supplementary Figure S1). To confirm the 35S:amiR-*SlBOP1-3* transgene silencing effect, mRNA levels of the three tomato *SlBOP* gene members (*SlBOP1, SlBOP2*, and *SlBOP3*) were analyzed and compared to wild type (WT). A significant decrease in *SlBOP1, SlBOP2*, and *SlBOP3* transcript was observed. *SlBOP2* and *SlBOP3* expression was decreased to less than 40% of WT levels, while *SlBOP1* transcript was reduced to 60% of WT levels (Supplementary Figure S2). To study the impact of *SlBOP* reduced expression on the axil phenotype, we utilized High Resolution Episcopic Microscopy (HREM ((Weninger and Mohun, 2007; Pokhrel et al., 2017) to create three dimensional (3D) reconstructions of WT and 35S:amiR-*SlBOP1-3* leaf axils (**Figure 1**). Altered morphology of the 35S:amiR-*SlBOP1-3* leaf axil in comparison to WT, was evident in the HERM 3D images. While in WT plants the AM was nested in the leaf axil at the junction between the stem and the leaf petiole (**Figures 1A,C,E**), in 35S:amiR-*SlBOP1-3* plants the AM was displaced and shifted along the proximal-distal axis to be positioned on the adaxial side of the leaf petiole (**Figures 1B,D,F**). To further study the role of SlBOP in leaf axil patterning, the stem-petiole junctions of WT, 35S:amiR-*SlBOP1-3* and triple mutant *CR-slbop1/2/3* plants were analyzed using Scanning Electron Microscopy (SEM). Both 35S:amiR-*SlBOP1-3* and *CR-slbop1/2/3* plants exhibited an abnormal leaf axil phenotype consisting of a dislocated AM on the leaf petiole, in contrast to WT plants that displayed the AM at the petiole base attached to the stem (**Figures 2A–C**). In-depth analysis of the mutant plant phenotypes showed that the *CR-slbop1/2/3* plants exhibited a variety of leaf axil phenotypes characterized by changes in AM positioning and ranging from a phenotype similar to that of the 35S:amiR-*SlBOP1-3* plants to more severe phenotypes with AM positioning further displaced and shifted toward the abaxial side of the leaf petiole (**Figure 2C**). To assess the frequency of the AM abnormal positioning in the BOP-silenced and mutant plants, 50 plants from each genotype; 35S:amiR-*SlBOP1-3*, triple mutant *CR-slbop1/2/3* or WT plants, were scored for AM positioning. The first leaf of 4 week old plants was detached from the stem, and AM location, either attached to the stem or removed with the detached leaf petiole, was determined using a dissecting scope. While in 96% of the 35S:amiR-*SlBOP1-3* transgenic plants and 94% of the triple mutant *CR-slbop1/2/3* plants, the AM was located on the detached petiole due to AM displacement, 94% of the WT AMs remained attached to the stem. To compare the cellular morphology of the leaf axil in WT, 35S:amiR-*SlBOP1-3* and *CR-slbop1/2/3* plants, sections of the stem-petiole region were examined by light microscopy. The displacement of the AM was clearly observed in the 35S:amiR-*SlBOP1-3* and *CR-slbop1/2/3* plants relative to WT (**Figures 2D–F**). In addition, we noted that the cellular morphology of the 35S:amiR-*SlBOP1-3* and *CR-slbop1/2/3* leaf axils was altered. The small cells observed in WT, at the base of the leaf petiole distal to the AM, in the region where leaf abscission takes place in WT plants (**Figures 2D,G** and Supplementary Figures S3C–F), were absent in the 35S:amiR-*SlBOP1-3* and *CR-slbop1/2/3* plants (**Figures 2E,F,H,I**), In view of these results the effect of BOP silencing/abolishment on abscission was studied in detail.

**FIGURE 3:**
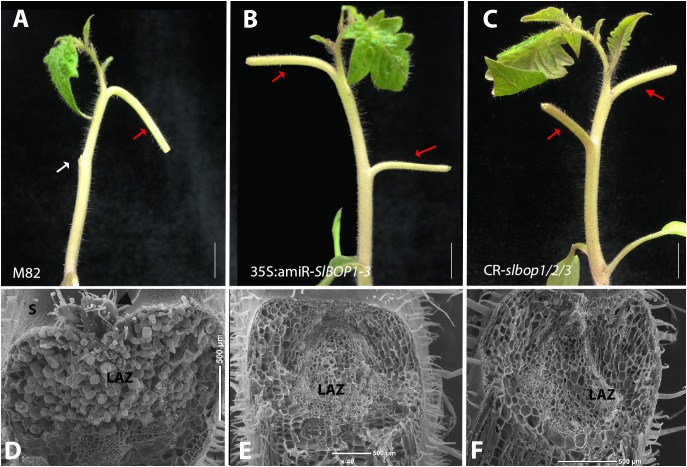
*SlBOP* downregulation and mutagenesis lead to abolishment of leaf abscission. The leaf blade was removed from the first leaves of 4-weeks old wild type (M82) **(A)**, 35S:amiR-*SlBOP1-3* **(B)** plants and *CR-slbop* triple mutant *CR-slbop1*/2/3 **(C)**. Following blade removal the plants were sprayed with ethephon to induce petiole abscission. **D–F**, SEM analysis of the leaf axil after petiole abscission at the first leaves of a 4-week old wild type **(D)**, 35S:amiR-*SlBOP1-3* **(E)** and *CR-slbop1*/2/3 plants **(F)**. White arrow – abscised petioles, red arrows – non-abscised petioles. LAZ, leaf abscission zone; S, stem; black arrowhead – axillary meristem. Bars **(A–C)** = 1 cm, **(D–F)** = 200 μm.

We first followed the leaf abscission process in tomato WT plants. Removal of the leaf blade, which serves as an abscission-inhibiting auxin source, followed by ethylene treatment leads to leaf abscission (Lers et al., 2006). To analyze the timing of leaf abscission in tomato we similarly induced leaf abscission by blade removal followed by ethephon application (metabolized in the plant to ethylene) in leaves of 3 and 4-week old plants. While the first leaf petiole of ethephon-treated 3-week old tomato plants remained attached to the stem, the first leaf petiole of 4-week old plants abscised in all the treated plants (Supplementary Figures S3A,B). In WT plants cell separation in the abscising leaves occurred at the base of the leaf petiole, distal to the AM (Supplementary Figures S3G,H) at a visible junction between small cells (on the proximal side) and elongated cells (on the distal side) (Supplementary Figures S3C–F). Thus, the AM remains attached to the plant body post-abscission (Supplementary Figures S3I). Petiole abscission was observed, after blade removal and ethephon treatment, in all the WT leaves (**Figure 3A**), while a complete inhibition of petiole abscission was observed in all the 35S:amiR-*SlBOP1-3* and *CR-slbop1/2/3* treated plants (**Figure 3B,C**). Lack of abscission in the BOP-silenced and mutant plants was tested over a length of time and detachment of the leaf petioles from the stem was only possible after application of significant force resulting in tissue tearing. Comparative analysis of the stem-petiole surface after leaf detachment revealed that the exposed cell surface in WT plants was composed of intact cells with round phenotype consistent with an active abscission process (**Figure 3D**), whereas the parallel tissue in the 35S:amiR-*SlBOP1-3* and *CR-slbop1/2/3* plants exhibited damaged and broken cells consistent with tearing due to the lack of cell separation processes (**Figures 3E,F**). Our results suggest that the tomato *SlBOP* gene family is involved in patterning of the leaf axil along the proximal-distal axis, determining the AM location at its base and controlling the adjacent leaf AZ development.

### *SlBOP2* Is the Dominant Gene in Leaf Axil Patterning

**FIGURE 4:**
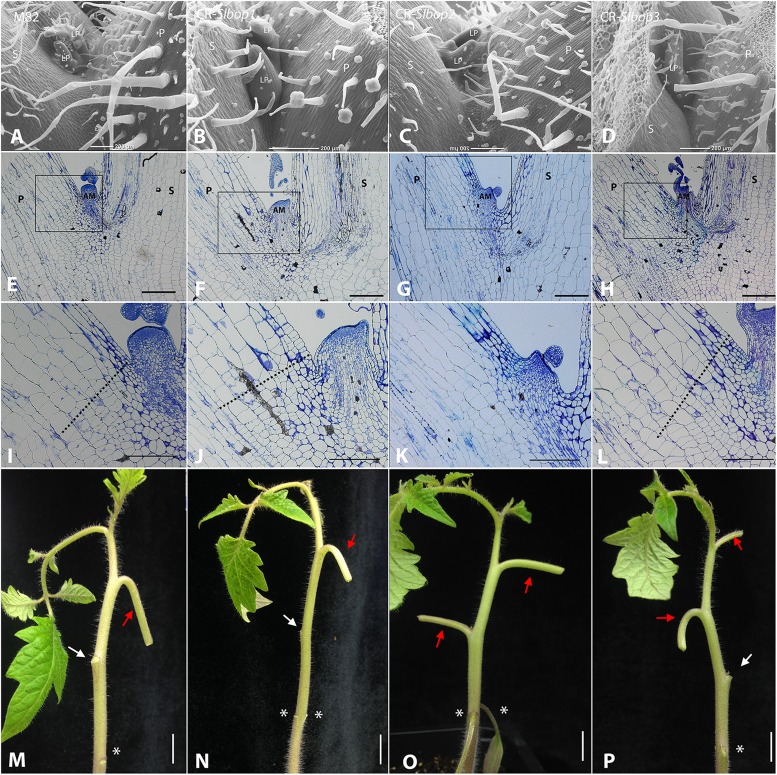
SlBOP2 is the main SlBOP involved in leaf axil patterning. **A–D**, SEM analysis of the first leaf axil of 4-week old wild type (M82) **(A)** and *CR-slbop* single mutant plants: *CR-slbop1* **(B)**, *CR-slbop2* **(C)**, and *CR-slbop3* **(D)**. **E–L**, Histologic analysis of the first leaf petiole of 4-week old wild type **(E, I)**, *CR-slbop1* **(F,J)**, CR-*slbop2* **(G,K)**, and CR-*slbop3* **(H,L)**. **I–L**, close up of the leaf axil framed in **E–H**, respectively. **M–P**, Leaf abscission was induced in the two first leaves of 4-week old wild type **(I)** *CR-slbop1* **(J)** *CR-slbop2* **(K)**, and *CR-slbop3* **(L)**. P, petiole; AM, axillary meristem; S, stem; LP, leaf primordium, white arrows – abscised petiole, red arrow – non-abscised petiole, asterisks – location of the cotyledon attachment to the stem. Bars **(A–H)** = 200 μm, **(I–L)** = 100 μm, **(M–P)** = cm.

To further investigate the functional divergence or redundancy between the three tomato *SlBOPs* in leaf axil patterning, we analyzed single gene editing based *CRISPR (CR)-slbop* null mutants, *CR-slbop1*, C*R-slbop2*, and *CR*-*slbop3*, for the leaf axil phenotype (Xu et al., 2016). SEM and histologic analysis of the *CR-slbop* single mutant plants showed that *CR-slbop1* and *CR-slbop3* exhibited a phenotype similar to WT, characterized by normal location of the AM at the leaf axil, nested between the base of the leaf petiole and the stem (**Figures 4A,B,D–F,H**). In contrast, the *CR-slbop2* leaf axil phenotype was abnormal, manifested by AM displacement on the adaxial side of the petiole (**Figures 4C,G**). The cellular morphology of the *CR-slbop2* leaf axil lacked small cells at the putative AZ (**Figure 4K**), while *CR-slbop1* and *CR-slbop3* exhibited a visible AZ characterized by a boundary between small cells on the proximal side and elongated cells on the distal side at the base of the leaf petiole (**Figures 4J,L**), similar to WT (**Figure 4I**). To further quantify AM displacement, the distance between the stem-petiole junction (the location of the AM in WT plants) and the actual location of the AM was measured and compared in the *CR-slbop2* and 35S:amiR-*SlBOP1-3* plants. The 35S:amiR-*SlBOP1-3* plants exhibited a significant difference in the AM displacement compared to the *CR-slbop2* plants (Supplementary Figure S4). Induced leaf abscission occurrence in the *CR-slbop* single mutants was consistent with the observed AZ cellular morphology; all *CR-slbop1* and *CR-slbop3* mutants abscised normally (**Figures 4N,P**), while abscission was abolished in all the *CR-slbop2* mutant (**Figure 4O**). To further study the functional divergence or redundancy between the SlBOP family members, we analyzed the double mutant combinations – *CR-slbop1/2, CR-slbop2/3*, and *CR-slbop1/3* – for the leaf axil phenotype and for induced leaf abscission. Proper AM location and leaf abscission were observed only in the double mutant *CR-slbop1/3* (Supplementary Figures S5C,G) but were impaired in the genetic combinations which included *CR-slbop2* (i.e., *CR-slbop1/2, CR-slbop2/3*) (Supplementary Figures S5B,D,F,H).

### TMF May Function in Concert With SlBOP to Regulate Leaf Axil Patterning, AM Positioning and AZ Development

**FIGURE 5:**
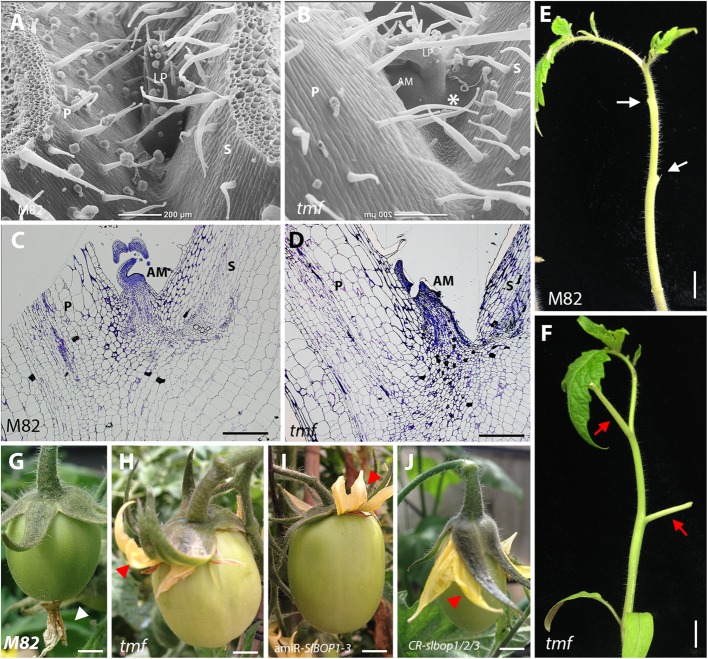
*tmf* leaf axil and leaf and floral organ abscission phenotypes. SEM analysis **(A,B)** and histologic analysis **(C,D)** of the first leaf axil of 4-week old wild type (M82) **(A,C)** and *tmf* **(B,D)** plants. **E,F**, Leaf abscission was induced in the two first leaves of 6-week old wild type **(E)** and *tmf* **(F)** plants. **G–J**, Abscission of the floral organs in wild type **(G)**, *tmf* **(H)**, 35S:amiR-*SlBOP1-3* **(I)**, and *CR-slbop1*/2/3 plants **(J)**. White arrow – abscised petiole, red arrow – non-abscised petiole, white triangle – abscised floral organs, red triangle – non-abscised floral organs. P, petiole, AM, axillary meristem, S, stem. Bars **(A–D)** = 200 μm, **(E–J)** = 1 cm.

All three tomato SlBOP proteins, SlBOP1, SlBOP2, and SlBOP3, have been shown to interact with TMF, a member of the ALOG family of proteins, to control flowering, meristem maturation and inflorescence architecture (MacAlister et al., 2012; Xu et al., 2016). To further investigate whether TMF interacts with SlBOPs to regulate leaf axil patterning and organ abscission, we analyzed *tmf* null mutant plants. The *tmf* mutants exhibited a range of leaf axil phenotypes characterized either by displacement of the AM to the adaxial side of the leaf petiole (**Figures 5A,B**), similar to *CR*-*slbop* single (CR-*slbop*2), double (*CR-slbop1/2, CR-slbop2/3)*, and triple (*CR-slbop1/2/3*) loss of function mutants and to 35S:amiR-*SlBOP1-3* (**Figures 1**, **2**, **4** and Supplemental Figure S5), or by fusion of the AM to the base of the leaf petiole (**Figures 5C,D**). Consistent with the visible effect on leaf axil patterning, *tmf* null mutant plants were also impaired in leaf petiole abscission following induction (**Figures 5E,F**), similar to *CR-slbop* mutant combinations which included the *CR-slbop2* and to the 35S:amiR-*SlBOP1-3* transgenic plants (**Figures 3**, **4** and Supplementary Figure S5). *tmf* mutant plants were also analyzed for floral organ abscission. In WT tomato plants abscission of the floral organs, the fused corolla and stamens, takes place at their base, following successful fertilization and initiation of fruit development (**Figure 5G**). Floral organ abscission was abolished in *tmf* mutant plants and the drying floral organs remained attached to the developing fruits (**Figure 5H**), similar to *CR-slbop1/2/3* and 35S:amiR-*SlBOP1-3* (**Figures 5I,J**). These results suggest that TMF is indeed involved in leaf axil patterning, AM positioning and leaf and floral organ abscission.

## Discussion

Leaf axil patterning along the proximal-distal axis begins as the leaf primordium emerges from the peripheral zone of the SAM and a new BZ is established between the SAM and the developing leaf primordium. Later in development the AM initiates at the BZ between the stem and the leaf base (Žádníková and Simon, 2014; Hepworth and Pautot, 2015; Wang et al., 2016). In Arabidopsis, *BOP* genes have been shown to be expressed early in leaf development at the incipient leaf primordium and in the proximal region of the emerging leaves marking the leaf primordium BZ (Norberg et al., 2005; Ha et al., 2007; Jun et al., 2010; Khan et al., 2014). Similarly, tomato SlBOP genes mark lateral organ boundaries and are expressed early during tomato leaf primordium development (Xu et al., 2016). In the present work we show that loss of function of *SlBOP*s, or downregulation of their expression by microRNA, altered the position of the AM while concomitantly abolishing leaf abscission and any visual features of the leaf AZ morphology (**Figures 1–3**). Moreover, specific gene knockout demonstrated that of the three *SlBOP* gene members, loss-of-function of *SlBOP2* alone was sufficient to cause AM dislocation and to abolish leaf abscission by altering cellular structure in the putative AZ (**Figure 4**), while loss of function of *SlBOP1* or *SlBOP3* did not cause any visual phenotype related to the leaf axil. These results suggest that SlBOP2 is the main regulator of leaf axil patterning along the proximal-distal axis, controlling boundary formation during leaf primordium development, specifying AM positioning and enabling AZ development. However, the enhanced AM dispositioning phenotypes observed in the 35S:amiR-*SlBOP1-3* and the triple *CR-slbop1/2/3* mutants, relative to *CR-slbop2* (**Figures 2B,C**, **4C**), suggests a limited effect of *SlBOP1* and *SlBOP3* on axil development, at least in the absence or low levels of *SlBOP2*. Thus, we cannot preclude the involvement of *SlBOP1* and *SlBOP3* in proper proximal-distal leaf axil patterning.

Lateral leaf BZs, located at the leaf axils between the SAM and the leaf primordium, are the sites for future development of the AM and leaf AZ (Žádníková and Simon, 2014). The present data suggest that SlBOPs act throughout leaf development to pattern the proximal-distal axis of leaf axils, starting early in development to promote the SAM-leaf primordium boundary identity, followed by marking the AM-leaf petiole boundary, and later controlling leaf AZ formation, which differentiates at the boundary location. Hence, impaired expression of *SlBOPs* leads to abnormal positioning of the meristem as well as to loss of cellular configuration in the leaf AZ, characterized by the loss of typical juxtapositioning between dividing and elongating cells. While the data support the notion that AM positioning and AZ formation are both directly regulated by BOP, we cannot rule out the possibility that loss of leaf AZ differentiation is not directly related to loss of SlBOP expression, but rather is the outcome of the AM relocation.

SlBOP-TMF complexes have been shown to be involved in tomato flowering and inflorescence development (Xu et al., 2016). Our results show that TMF, similar to SlBOPs, is involved in leaf axil patterning along the proximal-distal axis, suggesting that TMF-SlBOP complexes may also play a role during tomato vegetative development. BOP proteins display high functional diversity in regulating a large number of developmental processes in flowering plants, such as leaf patterning, flower development, hypocotyl elongation, leaf and flower abscission, nectary, stipule, and nodule development (Ichihashi et al., 2014; Khan et al., 2014). However, the molecular framework enabling BOP protein family members to regulate these various responses and exert specificity is poorly understood. It was suggested that BOP proteins, similar to NPR1, interact through the conserved ankyrin repeats and BTB/POZ with other TFs to regulate downstream targets. Different BOP-interacting proteins may generate the complexity required to regulate diverse transcriptional targets. Indeed, Xu et al. (2016) showed that two TMF homologs, TFAM1 (TMF Family Member) and TFAM2 are able to interact with SlBOPs and their respective mutants exhibit loss of function phenotypes resembling those of *CR-slbops* mutants. *CR-tmf1* is characterized by inhibition of floral organ abscission, and abnormal fruit shape, similar to the *CR-slbop1, CR-slbop2*, and *CR-slbop3* single mutants, and fused reproductive organs that match the triple *CR-slbop1/2/3* phenotype, while *CR-tfam2* shows inflorescences with single branching often observed in *CR-slbop2* single mutants. Thus, the accumulating data suggest the presence of different SlBOP transcriptional enhancing complexes involving a variety of interactors. Deciphering the members of these putative complexes, as well as their downstream targets, will enable further understanding of SlBOP roles during plant development.

## Materials and Methods

### Plant Materials and Growth Conditions

All Tomato (*Solanum lycopersicum*) plants were in the M82 cultivar background. Plants were grown in growth chambers for 4 weeks under 16 h of cool-white fluorescent light with temperature ranging between 23 and 26°C. After 4 weeks the plants were moved to the greenhouse and grown under natural light with temperatures ranging between 18 and 25°C.

An artificial miR designed to downregulate *SlBOP1-3* genes (amiR-*SlBOP1-3)* was constructed as described in Supplementary Figure S1, according to Alvarez et al. (2006), and cloned downstream of the 35S promoter of the pART7 vector. Constructs were then subcloned into the pMLBART binary plasmid. Tomato transformation was performed as described (McCormick, 1997). CRISPR/Cas9 (*CR*)-*slbop* mutants are described in Xu et al. (2016) and the *tmf* mutant is described in MacAlister et al. (2012).

Solyc *SlBOP* gene: *SlBOP1* (Solyc04g040220), *SlBOP2* (Solyc10g079460), *SlBOP3* (Solyc10g079750).

### Leaf Abscission Induction

Leaf abscission assay was carried out on the first and second leaves of 4-week-old plants, unless stated otherwise. Leaf abscission was induced by removal of the leaf blade at the blade-petiole junction with a sharp razor blade. Twenty four hours after blade removal the plants were sprayed with 4 mM ethephon (Bayer). Leaf petiole abscission or lack-of-abscission was scored 48 h after ethephon treatment (time point corresponding to 100% abscission in WT plants) and confirmed 1 week later. Twenty five to thirty plants were treated in a single leaf abscission experiment and five biological replicas were carried out for each experiment.

### Histologic Analysis

Histologic analysis was done to 10 plants from each genotype. Tissues were fixed overnight in 4% paraformaldehyde (PFA) (v/v), rinsed in PBS buffer and dehydrated through an ethanol series up to 95% ethanol. Fixed and dehydrated issues were infiltrated with catalyzed monomer A of the JB-4 embedding kit (Electron Microscopy Sciences) and embedded into JB-4 plastic resin under an oxygen-free environment. Blocks were serially sectioned (interval 4 mm) on a rotary microtome (Leica RM2255) with TC-65 disposable blades (Leica). Sections were stained with 0.1% (w/v) toluidine blue prior to examination and photographed on an Olympus BX53 digital microscope equipped with an Olympus DP73 digital camera using bright field.

### Scanning Electron Microscopy (SEM) Analysis

Fifteen plants from each genotype were analyzed using SEM. Tissues were fixed in 70% ethanol overnight and dehydrated through an ethanol series up to 100% ethanol. Tissues were dried at the critical point, mounted on a metal stub and coated with gold. Scanning electron microscopy was performed using JCM-6000 Benchtop SEM (JEOL).

### Quantification of AM Displacement in Tomato *CR-slbop2* and 35S:amiR-*SlBOP1-3* Plants

The distance between the stem-petiole junction (the location of the AM in WT plants) and the actual location of the AM was measured and compared in the *CR-slbop2* and 35S:amiR-*SlBOP1-3* plants. Measurements were done on 10 plants from each genotype by utilizing the light microscopy sections and SEM images. Data were analyzed using JMP pro 13 (SAS Institute Inc) and summarized by means and standard errors. Student’s *t*-test was performed (*p* ≥ 0.05).

### HREM Scanning

HREM analysis was done to four plants from each genotype. Tissues were fixed overnight in 4% paraformaldehyde (PFA) (v/v), rinsed in PBS buffer and dehydrated through an ethanol series up to 95% ethanol. Fixed and dehydrated tissues were infiltrated with catalyzed monomer A of the JB-4 embedding kit (Electron Microscopy Sciences) containing eosin and acridin orange dyes as previously described (Weninger and Mohun, 2007; Pokhrel et al., 2017). Samples were incubated for 7 days on a revolving shaker at room temperature in the dark. Samples were positioned and mounted in plastic molds and sectioned using the HREM system (Indigo Scientific, Baldock, United Kingdom). Images of >800 sections (2.5 μm thick) were captured, stacked, and processed using Fiji software (Schindelin et al., 2012) and 3D reconstruction was performed using Amira software (FEI, Hillsboro, OR, United States).

### Quantitative RT–PCR (qRT–PCR)

RNA was extracted from five leaf AZs for each biological replicate with a Plant/Fungi Total RNA Purification kit including DNase I treatment (Norgen Biotek Corp.). Total RNA (0.5–1 μg) was used for cDNA synthesis with an All-In-OneRT MasterMix (Applied Biological Materials Inc.). qRT–PCR was performed using gene-specific primers (Supplementary Table S1) and Fast SYBR^®^ Green Master Mix (Applied Biosystems) using the Stepone Plus^TM^ real time PCR system (Applied Biosystems) following the manufacturer’s instructions. qRT-PCR analysis was normalized to the reference gene Solyc04g064820 and/or Solyc03g111090 selected according to Ichihashi et al. (2014).

## Author Contributions

RL-A performed the quantitative RT–PCR analysis. JPA designed and constructed the artificial miR (amiR-*SlBOP1-3*) and generated the transgenic plants. YC and OG conducted the HERM analysis. AI and YE designed the experiments and wrote the manuscript. AI performed the experiments. All authors have read and approved the final version of the manuscript.

## Conflict of Interest Statement

The authors declare that the research was conducted in the absence of any commercial or financial relationships that could be construed as a potential conflict of interest.
