# Supplementary material for: The Tomato BLADE ON PETIOLE and TERMINATING FLOWER Regulate Leaf Axil Patterning Along the Proximal-Distal Axes
Source: Front Plant Sci. 2018 Aug 6;9:1126. doi: 10.3389/fpls.2018.01126 (PMC6087763; doi:10.3389/fpls.2018.01126)
Supplement: Supplementary file 1 [file Image_1.PDF]

(A)

amiR-*SlBOP1-3* (*At miR-164b* backbone)

```
CTCGAGGAGAATGATGAAGGTGTGTGATGAGCAAGATCTAGCGGCTGAGAGTGTATtTTACTAGCTC
ATATATACACTCTCACCACAAATGCGTGTATATATGCGGAATTTTGTGATATAGATGTGTGTGTG
TTGAGTGTGATGATATGGATGAGTTAGTTCatTACACTCTCttCCGCaAGATCATGACCACTCCACC
TTGGTGACGATGACGACGAGGGTTCAAGTGTTACGCACGTGGGAATATACTTATATCGATAAACACA
CACGTGCGGGATCC
```

```
10        20        30        40              50        60        70
|    A    CU         UUUU          A    ----   U    - -    A      G     A     A
GAUCU GCGG  GAGAGUGUA    ACUAGCUCAU UAUA    CAC CUCA C CACA AUGCGU UAUAU UGCGG A
CUAGA CGCC  CUCUCACAU    UGAUUGAGUA GUAU    GUG GAGU G GUGU UGUGUA AUAUA GUGUU U
^    A    UU         UACU          G    AGUA   U    U U    G      G     -     U
150       140       130       120       110       100        90         80
```

(B)

```
Solyc04g040220/SlBOP1 5′ GAUACACUCUCAGCCGCUAGA 3′
Solyc10g079460/SlBOP2 5′ GAUACACUCUCAGCCGCUAGA 3′
Solyc10g079750/SlBOP3 5′ GAUACACUCUCAGCCGCUAGA 3′
amiR-SlBOP1-3         3′ UUAUGUGAGAGUCGGCGAUCU 5′
```

Supplementary Figure 1: (A) The pre-amiR-*Sl BOP1-3* sequence and predicted fold-back structure in the Arabidopsis *(At) miR-164b* backbone with the *amiR-SlBOP1-3* sequence highlighted in red. (B) An alignment of the amiR-*SlBOP1-3* sequence with the RNA target sequences of the *SlBOP1-3* genes. G-U wobbles between the amiR-*SlBOP1-3* and the target sequences are marked in blue.
