# Supplementary material for: The Tomato BLADE ON PETIOLE and TERMINATING FLOWER Regulate Leaf Axil Patterning Along the Proximal-Distal Axes
Source: Front Plant Sci. 2018 Aug 6;9:1126. doi: 10.3389/fpls.2018.01126 (PMC6087763; doi:10.3389/fpls.2018.01126)
Supplement: Supplementary file 2 [file Image_2.PDF]

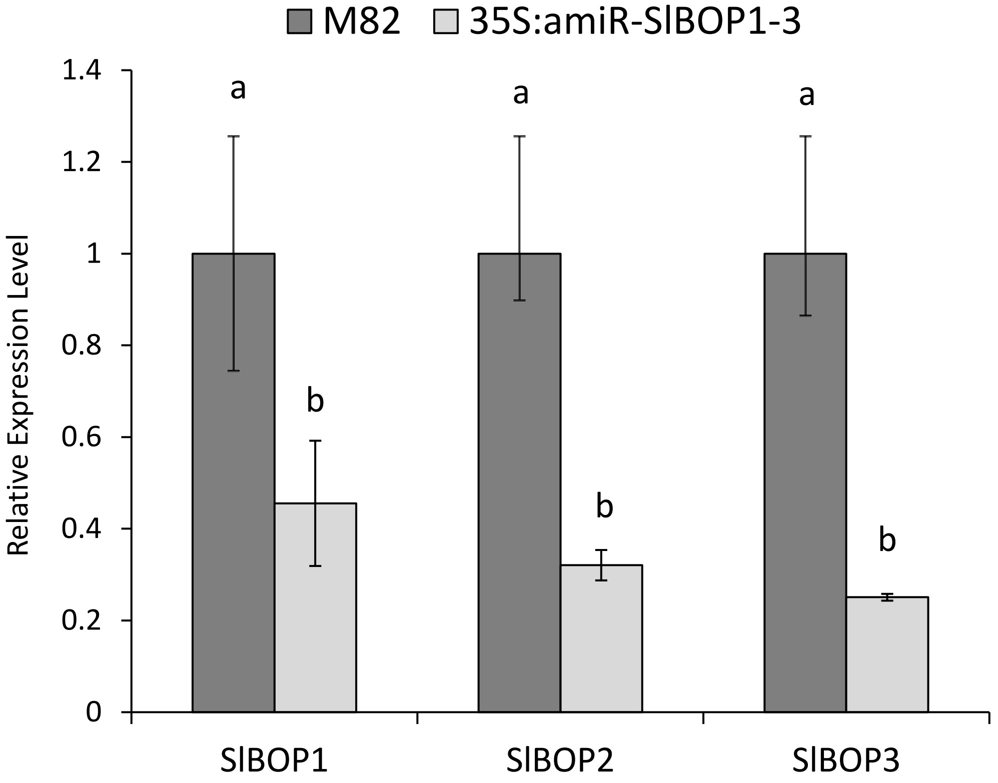

Supplementary Figure 2: Quantitative RT-PCR analysis. Quantitative RT-PCR analysis of *SlBOP1*, *SlBOP2* and *SlBOP3* mRNA levels in the leaf axils of the oldest leaves of four-week old wild type (M82) and 35S:amiR-*SlBOP1-3* transgenic tomato plants. Different letters indicate significant differences in the gene expression level between M82 and 35S:amiR-*SlBOP1-3* based on contrast t-test ($p \leq 0.05$).
