# Supplementary material for: The Tomato BLADE ON PETIOLE and TERMINATING FLOWER Regulate Leaf Axil Patterning Along the Proximal-Distal Axes
Source: Front Plant Sci. 2018 Aug 6;9:1126. doi: 10.3389/fpls.2018.01126 (PMC6087763; doi:10.3389/fpls.2018.01126)
Supplement: Supplementary file 3 [file Image_3.PDF]

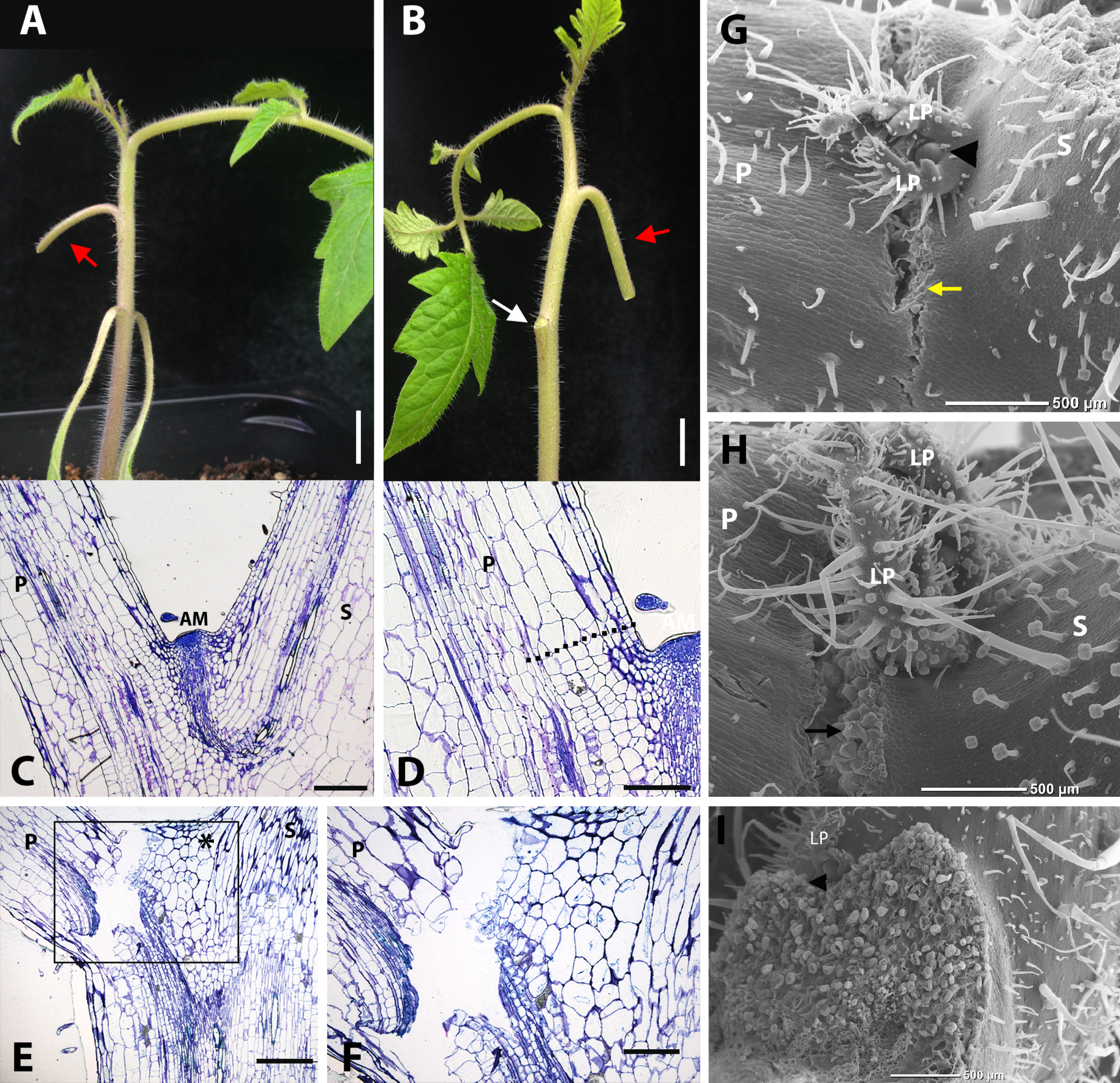
A
B
G
LP
LP
P
S
500 µm
H
LP
P
LP
S
500 µm
C
P
AM
S
D
P
AM
E
P
S
F
P
I
LP
500 µm

Supplementary Figure 3: Induction of leaf abscission in four-week old wild type tomato plants. A – B. Leaf blades were removed from the first leaves of three-week old (A) and four-week old (B) tomato plants. Following blade removal the plants were sprayed with ethephon to induce petiole abscission. C – F. Histologic analysis of the first leaf axil of a four-week old plant before (C-D) and after (E-F) ethephon treatment. D, F, close up of the area framed in C, E, respectively. Dotted line in (D) marks the junction between small cells and elongated cells. G – I. SEM analysis of the first leaf axil of a four-week old wild type (M82) tomato plants, after blade removal and ethephon spray. P - petiole, S - stem, AM - axillary meristem, LP – leaf primordium. White arrow – abscised petiole, red arrows – non-abscised petioles, yellow arrow – abscission zone, black arrow – cell surface after abscission, black arrowhead – axillary meristem, Bars (A-D) = 1 cm, (C, E) = 200 µM, (D, F) = 100 µM, (G-I) = 500 µM.
