# Supplementary material for: The Tomato BLADE ON PETIOLE and TERMINATING FLOWER Regulate Leaf Axil Patterning Along the Proximal-Distal Axes
Source: Front Plant Sci. 2018 Aug 6;9:1126. doi: 10.3389/fpls.2018.01126 (PMC6087763; doi:10.3389/fpls.2018.01126)
Supplement: Supplementary file 4 [file Image_4.pdf]

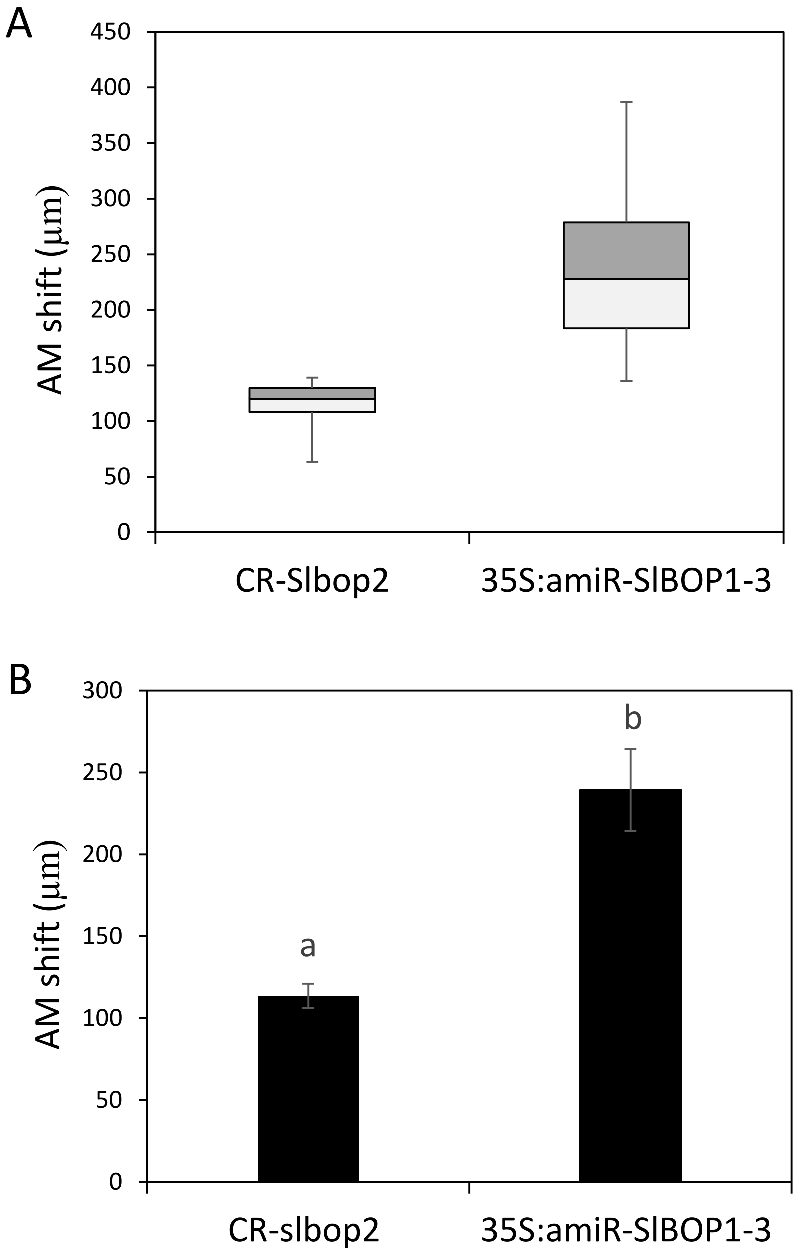
A
AM shift (µm)
450
400
350
300
250
200
150
100
50
0
CR-Slbop2
35S:amiR-SlBOP1-3
B
AM shift (µm)
300
250
200
150
100
50
0
a
b
CR-slbop2
35S:amiR-SlBOP1-3

Supplementary Figure 4: Axillary Meristem (AM) displacement in tomato 35S:amiR-*SlBOP1-3* and *CR-slbop2* plants relative to the AM position in wild type plants at the stem-petiole junction. (A) Box plot presentation of AM displacement in *CR-slbop2* and 35S:amiR-*SlBOP1-3* plants. (B) Histogram presentation of AM displacement in *CR-slbop2* and 35S:amiR-*SlBOP1-3* plants. Letters indicate significant differences in the AM shift between the *CR-slbop2* and 35S:amiR-*SlBOP1-3* plants based on student's t-test ($P \leq 0.05$).
