# Supplementary material for: The Tomato BLADE ON PETIOLE and TERMINATING FLOWER Regulate Leaf Axil Patterning Along the Proximal-Distal Axes
Source: Front Plant Sci. 2018 Aug 6;9:1126. doi: 10.3389/fpls.2018.01126 (PMC6087763; doi:10.3389/fpls.2018.01126)
Supplement: Supplementary file 5 [file Image_5.pdf]

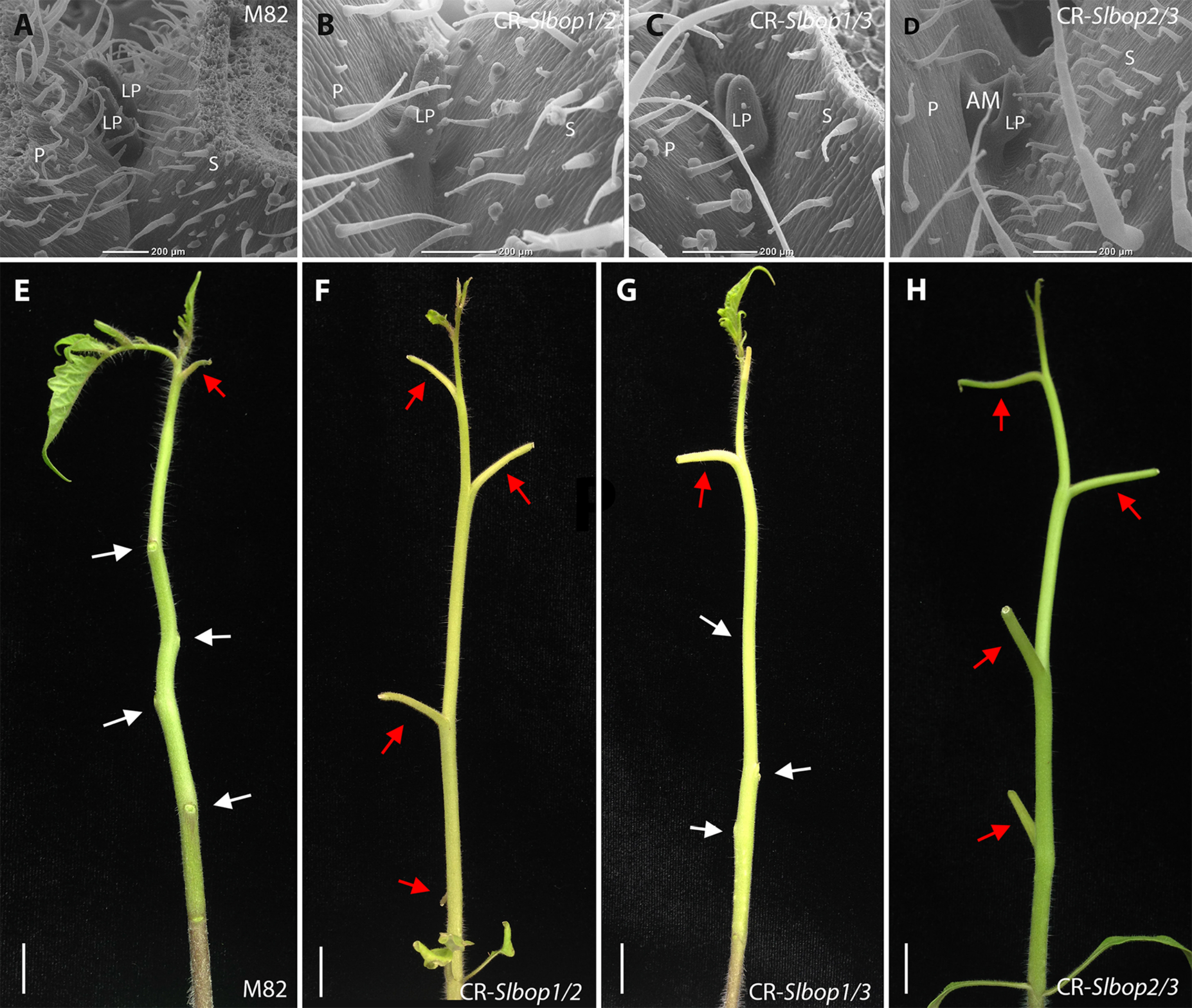

A
M82
LP
LP
P
S
200 μm
B
CR-Slbop1/2
P
LP
S
200 μm
C
CR-Slbop1/3
P
LP
S
200 μm
D
CR-Slbop2/3
P
AM
LP
S
200 μm
E
M82
F
CR-Slbop1/2
G
CR-Slbop1/3
H
CR-Slbop2/3

Supplementary Figure 5: Leaf axil patterning in tomato *Slbop* double mutant plants. A - D. SEM analysis of the first leaf axil of four-week old wild type (M82) (A) and *CR-slbop* double mutant tomato plants: *CR-slbop1/2* (B), *CR-slbop1/3* (C) and *CR-slbop1/3* (D). E – H. Leaf abscission was induced in the first four leaves of seven-week old wild type (E), *CR-slbop1/2* (F) *CR-slbop1/3* (G) and *CR-slbop2/3* (H). P- petiole, S – stem, LP – leaf primordium, white arrows – abscised petiole, red arrow – non-abscised petiole. Bars (A-D) = 200 µM, (F-I) = 1 cm.
