# Supplementary material for: The Tomato BLADE ON PETIOLE and TERMINATING FLOWER Regulate Leaf Axil Patterning Along the Proximal-Distal Axes
Source: Front Plant Sci. 2018 Aug 6;9:1126. doi: 10.3389/fpls.2018.01126 (PMC6087763; doi:10.3389/fpls.2018.01126)
Supplement: Supplementary file 6 [file Table_1.PDF]

Target gene	Forward primer (5'->3')	Reverse primer (5'->3')	Amplicon size (bp)
Solyc04g064820	ATAAGCCTGGGCTAGTGCAA	ACACATTCTCAACCCCCTTG	135
Solyc03g111090	ATATCGGTGGGAATGATCCA	TATCAGCATCCGATTCACCA	174
SlBOP1 (Solyc04g040220)	GTCCTTCCACATCTCTTTAATGAG	CTGTTAGGTCATTTTTAACTTG GAC	143
SlBOP2 (Solyc10g079460)	GGTAATTTCACGAGAGGAGGGT	TCCACTGCTCGTACTCGAACT	100
SlBOP3 (Solyc10g079750)	ATTCAAAGGTGCGATCCCTG	GTGGAAGATGGGACGTTGTTT G	132

Supplementary Table 1: Primers used for qRT–PCR.
